# AmpliSeq transcriptome analysis of human alveolar and monocyte-derived macrophages over time in response to *Mycobacterium tuberculosis* infection

**DOI:** 10.1371/journal.pone.0198221

**Published:** 2018-05-30

**Authors:** Audrey C. Papp, Abul K. Azad, Maciej Pietrzak, Amanda Williams, Samuel K. Handelman, Robert P. Igo, Catherine M. Stein, Katherine Hartmann, Larry S. Schlesinger, Wolfgang Sadee

**Affiliations:** 1 Center for Pharmacogenomics, Department of Cancer Biology and Genetics, College of Medicine, The Ohio State University, Columbus, Ohio, United States of America; 2 Texas Biomedical Research Institute, San Antonio, Texas, United States of America; 3 Division of Gastroenterology, Department of Internal Medicine, School of Medicine, University of Michigan, Ann Arbor, Michigan, United States of America; 4 Department of Population & Quantitative Health Sciences, Case Western Reserve University, Cleveland, Ohio, United States of America; 5 Center for Proteomics & Bioinformatics, Tuberculosis Research Unit, Case Western Reserve University, Cleveland, Ohio, United States of America; 6 European Organization for the Research and Treatment of Cancer, Brussels, Belgium; Institut de Pharmacologie et de Biologie Structurale, FRANCE

## Abstract

Human alveolar macrophages (HAM) are primary bacterial niche and immune response cells during *Mycobacterium tuberculosis* (*M*.*tb)* infection, and human blood monocyte-derived macrophages (MDM) are a model for investigating *M*.*tb*-macrophage interactions. Here, we use a targeted RNA-Seq method to measure transcriptome-wide changes in RNA expression patterns of freshly obtained HAM (used within 6 h) and 6 day cultured MDM upon *M*.*tb* infection over time (2, 24 and 72 h), in both uninfected and infected cells from three donors each. The Ion AmpliSeq™ Transcriptome Human Gene Expression Kit (AmpliSeq) uses primers targeting 18,574 mRNAs and 2,228 non-coding RNAs (ncRNAs) for a total of 20,802 transcripts. AmpliSeq^TM^ yields highly precise and reproducible gene expression profiles (R^2^ >0.99). Taking advantage of AmpliSeq’s reproducibility, we establish well-defined quantitative RNA expression patterns of HAM *versus* MDM, including significant *M*.*tb-*inducible genes, in networks and pathways that differ in part between MDM and HAM. A similar number of expressed genes are detected at all time-points between uninfected MDM and HAM, in common pathways including inflammatory and immune functions, but canonical pathway differences also exist. In particular, at 2 h, multiple genes relevant to the immune response are preferentially expressed in either uninfected HAM or MDM, while the HAM RNA profiles approximate MDM profiles over time in culture, highlighting the unique RNA expression profile of freshly obtained HAM. MDM demonstrate a greater transcriptional response than HAM upon *M*.*tb* infection, with 2 to >10 times more genes up- or down-regulated. The results identify key genes involved in cellular responses to *M*.*tb* in two different human macrophage types. Follow-up bioinformatics analysis indicates that approximately 30% of response genes have expression quantitative trait loci (eQTLs in GTEx), common DNA variants that can influence host gene expression susceptibility or resistance to *M*.*tb*, illustrated with the *TREM1* gene cluster and *IL-10*.

## Introduction

Susceptibility to tuberculosis (TB) varies substantially between individuals. While both genetic and environmental factors play essential roles in an individual’s susceptibility to *M*.*tb* infection or active TB [1–10], candidate gene and genome-wide association studies (GWAS) indicate the presence of single nucleotide polymorphisms (SNPs) in host genes that affect susceptibility to TB [1–8, 11]. Twin studies estimate the heritability (proportion of the variation in susceptibility attributed to genetics) to be as high as 80% but estimates vary over a substantial range [12]. The vast majority of candidate SNPs likely affects regulatory mechanisms, leading to a growing consensus that gene regulation plays a prominent role, particularly in genes subject to evolutionary selection pressures [7–9, 13].

Transcriptome analysis is used as a primary approach to assess the host cellular response to *M*.*tb* infection [14–27]. Whole transcriptomes have been measured with hybridization arrays and sequencing methods (RNA-Seq), providing a wealth of information, but these methods are either limited by a narrow dynamic expression range or yield substantially variable results, or both. RNA-Seq requires normalization to transform raw reads into relative expression levels which can introduce analytical artifacts [28, 29]. Here, we use next-generation RNA sequencing targeting RNA regions with specific primers (AmpliSeq Whole Transcriptome Human Gene Expression, 20,802 transcripts). This approach is reported to yield highly reproducible transcriptomes [30]. We apply AmpliSeq for the first time to measure transcriptomes in *M*.*tb-*infected MDM and HAM.

The precision and dynamic range of AmpliSeq [30] facilitates comparison of whole transcriptomes between different cell types. In this report, AmpliSeq is used to assess the degree to which MDM recapitulate the biological responses seen in freshly obtained HAM. This is a crucial practical question because whole blood is broadly available while obtaining bronchoalveolar lavage (BAL) to isolate HAM can be difficult and invasive. To account for substantial changes in RNA expression profiles over the incubation time, we use matching uninfected control samples and infected cells at 3 time points (2, 24, and 72 h). This experimental design accounts for differences in both tissue origin and *in vitro* culture conditions of HAM and MDM, with HAM used within 6 h of BAL whereas MDMs are derived from monocytes over several days in culture.

The resultant RNA expression profiles serve to identify differentially expressed (DE) genes at each time point following *M*.*tb* infection and characterize differences in RNA expression and pathways between MDM and HAM. To explore whether genetic factors contribute to the substantial inter-individual variability in *M*.*tb-*macrophage interactions, we scan DE genes for evidence of genetic variants affecting expression, with use of existing large-scale databases (such as GTEx, 1,000 genomes and dbGaP). Going into further detail, we focus on *IL-10* and *TREM1* as examples of how to integrate genetics with the transcriptome analysis.

## Materials and methods

### Collection and isolation of MDM and HAM

Fresh HAM and peripheral blood mononuclear cells (PBMCs) were obtained from tuberculin skin test (TST)-negative healthy human donors, under an approved IRB protocol at the OSUWMC, employing established protocols [31, 32]. HAM and PBMC samples were obtained from different donors. Written consent was obtained from each donor. MDM cultures were generated as described [31, 32]. Briefly, heparinized blood was layered on a Ficoll-Paque cushion to allow for collection of PBMCs which were cultured in RPMI (Life Technologies) with 20% autologous serum in Teflon wells (Savillex) for 5 days at 37°C/5% CO_2_ to allow for differentiation of monocytes into MDM. PBMCs were harvested and adhered to 24-well tissue culture plates (1.5x10^6^ PBMCs/well of which ~1.5x10^5^ were MDMs) for 2 h in RPMI with 10% autologous serum, and then lymphocytes were washed away to achieve purified MDM (> 99%) in monolayer culture. MDM monolayers were cultured for an additional 24 h before infection. For isolation and culturing of HAM from human BAL [33, 34], BAL samples obtained within 6 h were centrifuged and washed twice in PBS at 4°C, and the cell pellet was re-suspended in a minimum volume of RPMI medium. A portion of the cell suspension was subjected to cytospin followed by staining and microscopy to evaluate the percentage of macrophages in BAL (> 98%). After counting on a hemocytometer, the HAM were cultured in 24-well plates (1.5x10^5^ cells/well) in RPMI containing 10% human AB serum and Penicillin G (10,000 U/ml) for 2 h before washing the adhered cell monolayer and antibiotic removed. Single cell suspensions of *M*.*tb* H_37_R_v_ were prepared as previously described [35] and used for infecting macrophages at a 2:1 multiplicity of infection (MOI) for 2 h (similar uptake by HAM and MDM). Extracellular bacteria were washed away and incubation of the infected cell monolayer was continued in accordance with the time points selected. Macrophages were harvested in Trizol reagent after 2, 24 and 72 h, along with corresponding uninfected cells at the same time points from the same individual, to account for changes in RNA control expression over time. RNA was prepared using the Directzol kit according to manufacturer instructions[30]. RNA yield was ~ 500 ng– 1 ug per well with RNA Integrity numbers >7.

### Ion Torrent sequencing using the AmpliSeq transcriptome gene expression kit

Ion Torrent sequencing libraries were prepared according to the AmpliSeq Library prep kit protocol, and as published [30]. Briefly, 10 ng of total RNA was reverse transcribed, the resulting cDNA was amplified for 12 cycles by adding PCR Master Mix, and the AmpliSeq human transcriptome gene expression primer pool (targeting 18,574 protein-coding mRNAs and 2,228 non-coding ncRNAs) (based on UCSC hg19). Amplicons were digested with the proprietary FuPa enzyme, then barcoded adapters were ligated onto the target amplicons. The library amplicons were bound to magnetic beads, and residual reaction components were washed off. Libraries were eluted and individually quantitated by qPCR using Ion Torrent P1 and A sequencing primers and SYBR Green master mix. Individual libraries were diluted to a 50 pM concentration, then combined in batches for further processing. Emulsion PCR, templating and PI chip loading was performed with an Ion Chef Instrument (Thermo-Fisher). Sequencing was performed on an Ion Proton ^TM^ sequencer (Thermo-Fisher), with HiQ sequencing chemistry. The analyses were performed in the Core Laboratory of the OSU Center for Pharmacogenomics, which is certified for AmpliSeq analysis by Thermo-Fisher.

### Transciptome analysis

AmpliSeq sequencing data were analyzed using the Ion Torrent Mapping Alignment Program (TMAP), as described [36]. To achieve both specificity and sensitivity, TMAP implements a two-stage mapping approach, using four alignment algorithms, BWA-short and long, SSAHA, and Super-maximal Exact Matching. This is followed by the Smith Waterman algorithm to find the final best mapping [37–40]. RNAs with less than ten read counts on average across all samples were excluded from further analysis. DE gene (DEG) analysis was performed with the R package edgeR [41]. The package is available from Bioconductor. DEGs were selected based on p-value and log2 fold change (log2FC).

For IPA analysis (https://analysis.ingenuity.com/), RNA expression levels were recorded as reads per million (RPM), normalized for the number of sequence reads per sample. Analysis of quantile distribution of RPM indicated a linear distribution between samples with different total reads occurring in a linear range, obviating the need for quantile normalization. DE RNAs were assessed at each time point for each individual sample as the ratio between RPM in *M*.*tb*-infected over control cells, using the mean of 3 measurements at each time point for MDM and HAM. Cutoff values of ≥2-fold change of mean RPM values between control and *M*.*tb*-infected cells, and adjusted p≤0.05 (estimated by edgeR) were used for selection of DE genes for the IPA analysis.

### Pathway analysis and bioinformatics

Baseline RNA levels were submitted to IPA. Predictions are based on statistical measures reflecting dataset genes known to interact with other proteins (overlap *p*-value) and activation (z-score) based on the known direction by which an upstream regulator influences its target (inhibition *versus* activation). In a similar fashion, DE genes (≥2-fold change, adjusted p≤0.05) were evaluated at each time point separately for MDM and HAM. Select genes were further tested for the presence of expression quantitative trait loci (eQTLs) in human blood (GTEx), for linkage disequilibrium (LD; 1,000 genomes project, http://archive.broadinstitute.org/mpg/snap/) and general GWAS hits (SNPs with genome-wide significance for clinical phenotpyes (Catalog, https://www.ebi.ac.uk/gwas/).

### Testing *IL-10* eQTL SNPs for association with TB disease

The sample for genetic association testing was from a household contact study in Kampala, Uganda; detailed recruitment and phenotyping methods have been described in our published reports [42, 43]. TB cases had active, culture-confirmed TB; controls had no evidence of active TB over two years of follow-up, although they may have had a positive TST. A total of 483 individuals (203 TB cases, 280 controls) were genotyped for the Illumina HumanOmni5 panel and passed quality control. Association testing was carried out under a logistic regression model, using generalized estimating equations (GEE) to correct for correlation due to family relationships, and adjusting for sex, HIV status and one principal component from a principal components analysis to adjust for population structure. An additive genetic model (on the log odds scale) for the number of minor alleles was assumed. The *IL-10* region was taken to be the GENCODE location of the *IL-10* gene (build GRCh37 of the human genome) plus 100 kilobase pairs upstream and downstream.

## Results

### Evaluation of AmpliSeq macrophage transcriptomes

**Fig 1:**
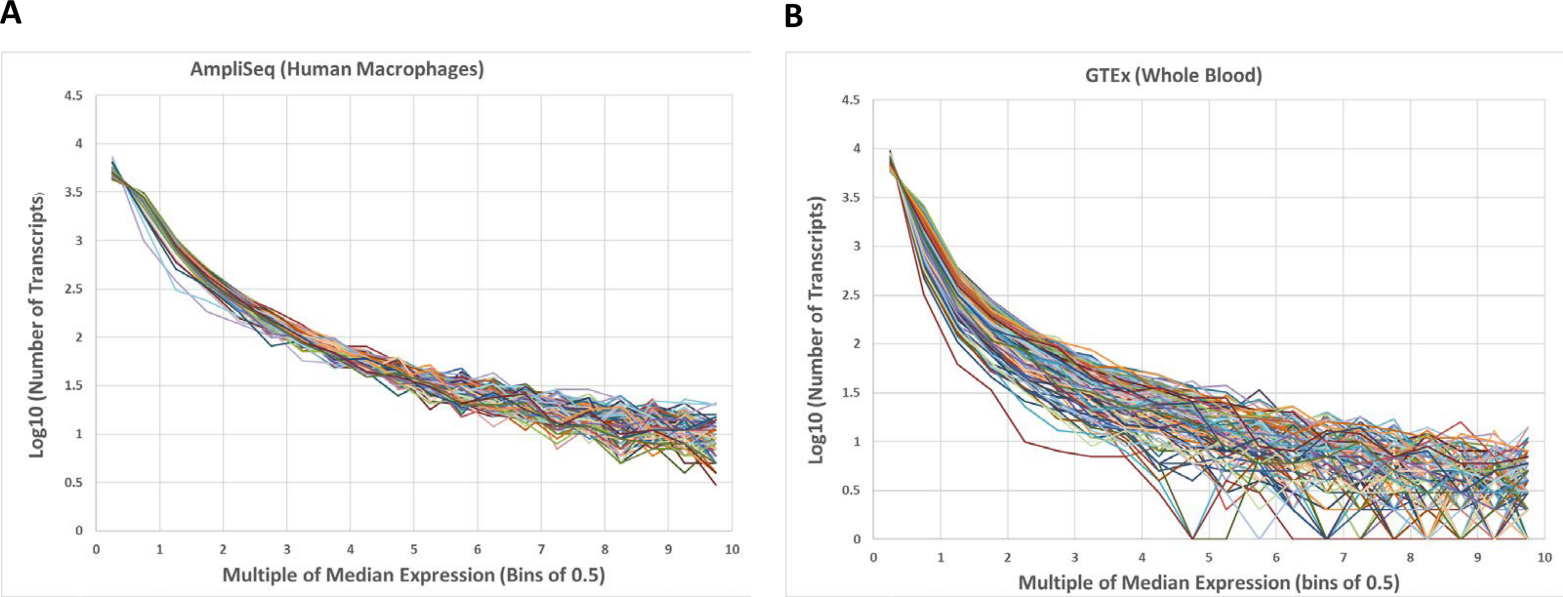
**Normalized AmpliSeq read count (RPM) distribution from 36 control and infected human macrophage (MDM and HAM) samples (A), compared to RNA-Seq counts from 36 randomly selected whole blood samples in GTEx (B)**. Note the large divergence at high RPM (high expression).

We performed AmpliSeq transcript analyses of MDM and HAM from three different donors each, in uninfected control and *M*.*tb-*infected cells at three time points (2, 24, and 72 h), totaling 36 samples. As a measure of precision and reproducibility, we compared the number of transcripts detected per sample, binning the results by AmpliSeq read counts. Fig 1 (panel A) displays the normalized count distribution from all 36 control and infected MDM and HAM samples employed in this study (panel A). Although the genes expressed differ to some extent between samples and conditions, the number of transcripts expressed in each given read count bin is nearly identical between samples for each bin between 1–10,000 RPM, with 11,271 transcripts detected, out of the total 22,804 targeted RNAs.

We further compared the AmpliSeq data obtained here from MDM and HAM with RNA-Seq results from an equal number of blood samples downloaded from GTEx (https://www.gtexportal.org/) [44] (Fig 1, panel B). The RNA-Seq data, obtained with poly-*dT* primers for cDNA production, are significantly dispersed at high RPM counts (panel B), in contrast to the AmpliSeq counts (panel A). For example, in some GTEx blood samples, only 3 genes are expressed at 6-fold the median level (Fig 1 panel B at a horizontal value of 6), while in other samples 30 genes are expressed at the same relative level. In contrast, AmpliSeq produces near quantile-normal measurements across all experiments regardless of infection status. Consequently, AmpliSeq transcriptome reproducibility enables robust detection of DE genes in a relatively small sample set, in particular for highly expressed genes, without the need for data normalization algorithms.

**Fig 2:**
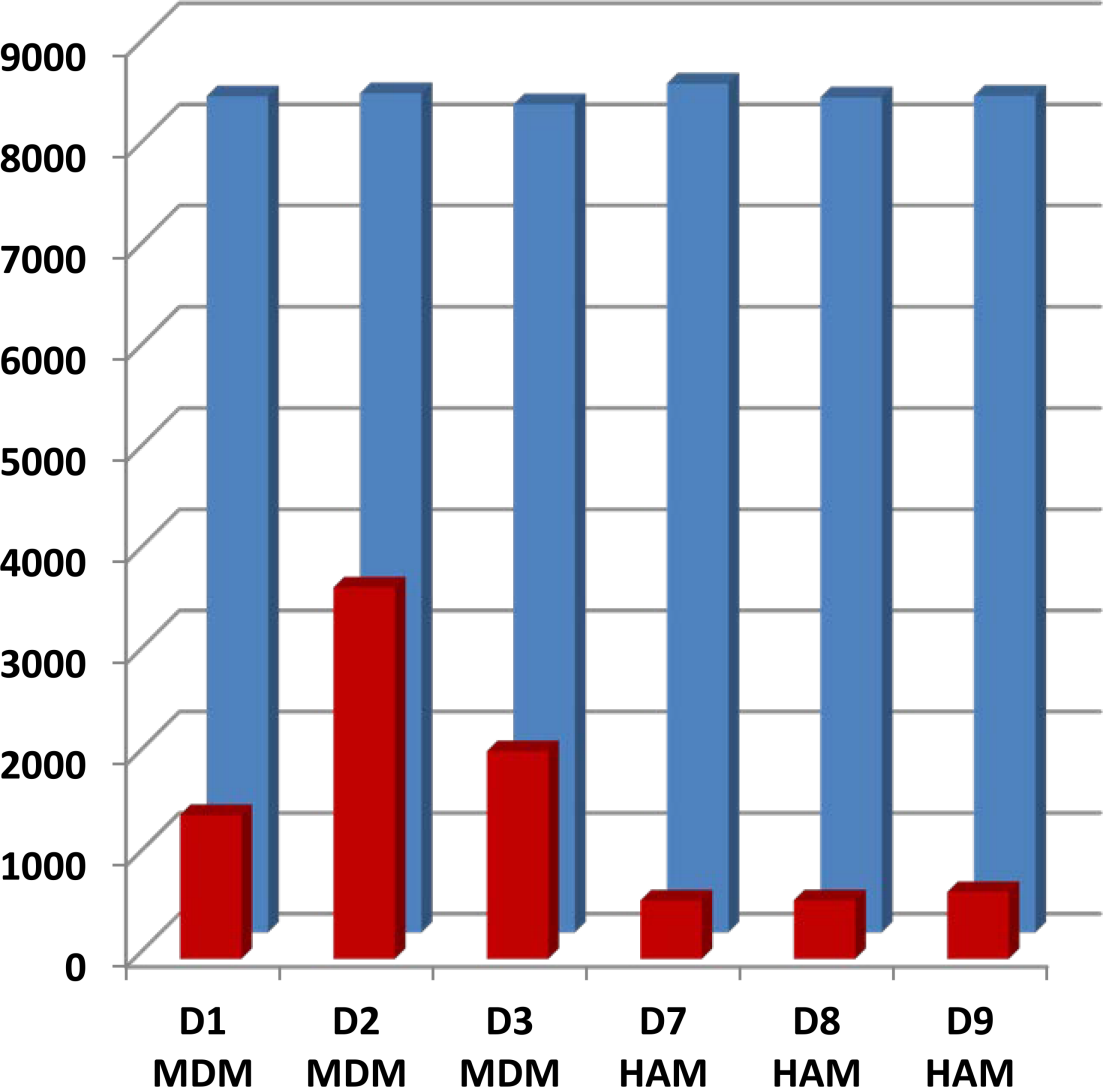
Comparison of transcript counts at read number >5 (RPM) and ≥1.5-fold (0.7 log2) differential expression ratio (DER) *M*.*tb*/control. Blue bars represent the total number of transcripts detected at 5 RPM or greater in individual 24 h control samples. Red bars indicate the number of transcripts differentially expressed at 0.7 log^2^ (≥1.5 fold in either direction) after 24 h exposure to *M*.*tb*. Note the increased number of DE transcripts in MDM compared to HAM.

A total of 11,271 gene transcripts were detected in all HAM and MDM combined. At a cutoff level >1 RPM, 9975–10301 different transcripts were detected in each individual sample, while at >5 RPM, the number of detected transcripts was just under 8,000 in all samples (Fig 2, blue bars). The number of detected transcripts was evenly distributed in all samples, throughout all count number bins. Replicates yielded nearly identical results with correlation coefficients of r^2^ = 0.9995±0.0001 for technical reproducibility chip to chip (n = 24 HAM), and r^2^ = 0.9994±0.0001 for independent replicate analyses of each sample (n = 14 duplicate HAM), confirming excellent reproducibility. Correlation coefficients between different wells and libraries form the same donor ranged from r^2^ = 0.92–0.99 (mean 0.972±0.028). Similar results were obtained with MDM and HAM. The AmpliSeq results will be made available in the NCBI Gene Expression Omnibus.

### Baseline RNA profiles of MDM and HAM

More than 11,000 RNA transcripts were detectable with AmpliSeq, over a range of 3–12 million reads per sample. The overall number of detectable RNAs does not significantly differ (p>0.05) between MDM and freshly obtained HAM, but individual genes differ in expression level between the two cell types. RNAs with prominent expression differences are listed in S1 Table. Genes expressed much higher in HAM than in MDM at 2 h include PTGS2, CXCL2, CXCL3, and TNFMDM. However, with continued *in vitro* culture of HAM, the high expression levels of these genes decline and approach levels observed in MDM at 72 h. Additional genes more robustly expressed in HAM at 2 h include PPARG, IL-8, and MARCO. Conversely, a number of genes are robustly expressed at 2 h only in MDM (MMP7, MMP9, CHI3L1, ITGA3), while in HAM their expression levels rise at 72 h, although not to the levels observed in MDM. A substantial number of these genes with large expression differences between HAM and MDM at 2 h has been associated with TB pathophysiology, asterisked in S1 Table. This analysis indicates that the resting states of cultured MDM and freshly obtained HAM differ substantially (2 h time point). However, over 72 h of *in vitro* incubation, HAM profiles become more similar to MDM. Notably, MDM are cultured for 5 days as part of the *in vitro* differentiation from monocytes, whereas the HAM cells are used ~ 6 h after harvesting of the BAL. Accordingly, comparisons between uninfected controls and *M*.*tb*-infected cells need to be performed at each time point.

Previously, the baseline gene expression profiles of HAM were shown to approximate more closely those of MDM (although the donor population differed having hemoptysis, the time of HAM use was not defined and culture conditions differed), except resting HAM initially displayed a more pro-inflammatory profile, with persistent up-regulation of components of the MHC Class II antigen presentation pathway [45]. In that study gene expression profiles also changed significantly over time. In another report, MDM and monocyte-derived DCs were shown to share 96% of the constitutively-expressed genes which include those associated with apoptosis, cell adhesion, chemotaxis, cytokines, cytokine receptors and surface molecules [21].

Ingenuity Pathway analysis (IPA) analysis of the AmpliSeq expression values shows that multiple baseline canonical pathways differ between the two cell types. Top IPA-derived canonical pathways in uninfected control MDM include: production of nitric oxide and reactive oxygen species in macrophages, TREM1 signaling, communication between innate and adaptive immune cells, and aryl hydrocarbon signaling. In contrast, top canonical pathways in uninfected control HAM are iCOS-iCOSL signaling in T Helper cells, Th1 pathway, IL-12 signaling and production in macrophages, and granulocyte adhesion and diapedesis.

While detailed analysis of baseline RNA profiles in MDM and HAM will require larger sample size than available in the present study, the AmpliSeq assay precision enables deep study of DE genes as we can compare each infected HAM sample to its own uninfected control at each time point, thereby enhancing ability to detect *M*.*tb-*induced host cell gene expression changes.

### *M*.*tb*-stimulated RNA profiles of MDM and HAM

**Fig 3:**
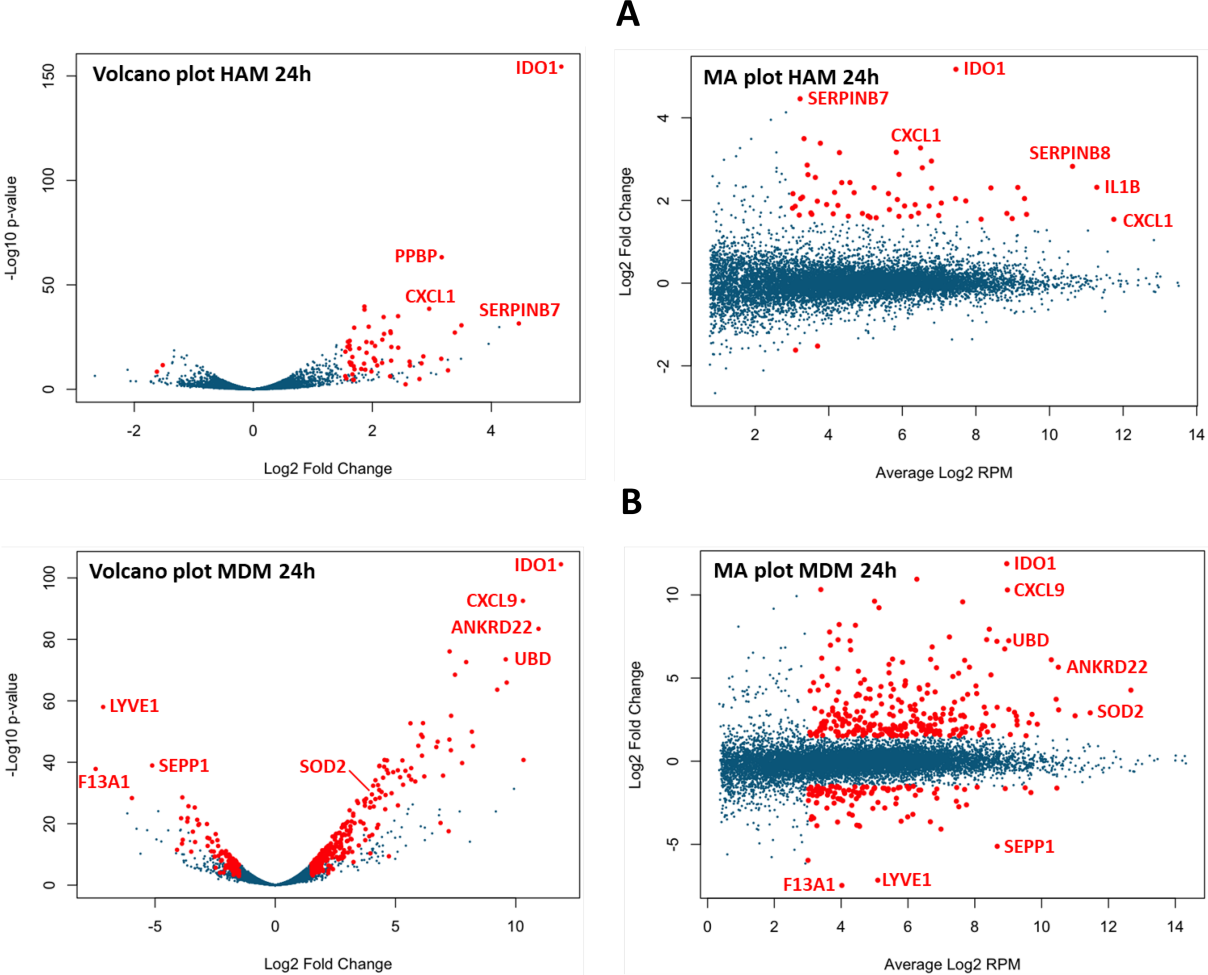
(**A)** Volcano plot (p value *versus* fold change ratio *M*.*tb*/control) and MA plot (fold change ratio *versus* average expression in RPM) of HAM at 24 h after *M*.*tb* infection. Red dots are significant at p = ≤0.05. A few genes are identified as shown, both up- and down-regulated. (**B)** The same plots for MDM at 24 h. Note the substantially greater response to *M*.*tb* compared to HAM. The number of unique DE genes is greater in MDM than HAM at all 3 time points. Fig 3A and 3B were generated from an interactive web site designed to display Volcano and MA plots of the HAM and MDM AmpliSeq data at all 3 time points; producing results tables with selectable expression, FDR and p-values. The site is accessible at https://dataportal.bmi.osumc.edu/macrophagedb/.

Infection with *M*.*tb in vitro* stimulates a more pronounced response in MDM compared to HAM. Shown in Fig 2, the number of genes nominally stimulated or repressed by *M*.*tb* by 1.5-fold or more (0.7 log2; DE genes; mean of 3 samples at each time point; red bars in Fig 2) is substantially greater in MDM compared to HAM. Applying a more rigorous statistical test using edgeR (adjusted p = ≤0.05), we find 16, 899, and 174 DE genes at 2, 24, and 72 h, respectively, in the 3 HAM donor samples, compared to 631, 1546, and 4489 DE genes, respectively, in MDM donor samples (S2 Table). These numbers show a similar trend compared to those obtained with the 1.5-fold DE threshold, with notably fewer genes recognized as significant at 2 h in HAM using edgeR. A searchable database of all RNAs detected by AmpliSeq in HAM and MDM is accessible at https://dataportal.bmi.osumc.edu/macrophagedb/, including a graphics interface. Entering thresholds of 1.5-fold DE, 8 or more RPM (log2–3), and adjusted p = ≤0.05 yields the Volcano and MA plots for the 2, 24, and 72 h time points. Representative plots of results at the 24 h time point are shown in Fig 3A and 3B. Sorted by expression level, fold-change upon *M*.*tb* exposure, and significance of stimulation, the graphs highlight differences between MDM and HAM.

The initial small response in HAM at 2 h is particularly noteworthy. The fold-change ratio *M*.*tb*/control at 2 h ranges from 0.9 to 3.1 in HAM and from 2.1 to 11.4 in MDM, with persistent differences observed at 24 h and 72 h. Among the 16 significant DE genes (adjusted p value ≤0.05) in HAM at 2 h, 11 are also DE genes with robust RNA ratios in MDM (CXCL3, CXCL2, CCL3, CCL20, PTX3, CSF2, IL-1B, JUN, IL-6, IER3, and SERPINB2). In contrast, IFNG, REXO1L2P, LINC00346, MT1L, and CRNKL1 are significant only in HAM at 2 h, with MT1L and CRNKL1 down-regulated by *M*.*tb*. On the other hand, many top scoring DE genes in MDM are missing in HAM (*e*.*g*., CXCL1, LOC440896, TNIP3, and SLC2A6). The top scoring DE genes include non-coding RNAs, for example, MIR155HG, LINC00346, LINC00515, LOC285972, and LOC283070 in HAM, and LOC440896, SNORA70G, LOC730101, LINC00641, LINC00113, and LOC145474 in MDM. Resolving the function of these ncRNAs requires future studies.

Between 24 h and 72 h post-infection, the number of DE genes (vs. uninfected control) rises in MDM; in contrast, there are fewer DE genes at the 72 h post-infection time point in HAM than in the 24 h post-infection time point (S2 Table). In HAM, many of the top scoring genes display lower expression in controls and less stimulation at 72 h compared to 24 h, suggesting factors of *in-vitro* incubation play a significant role in HAM; therefore, any *M*.*tb* effects on RNA expression require time-dependent controls. After an initial lag, at 72 h HAM show increased up-regulation of a greater variety of cytokines, interleukins, and other immune regulatory response molecules compared to MDM (S2 Table). In HAM, key genes include CCL20, CXCL9, IL-1B, IL-2A, IL-6, PTGS2, STAT1, and TNF at 72 h, while CCL5, CCL18, CXCL5, IL-1A, IL-6, IL-10, IL-32, PTGS2, and STAT3 stand out in MDM at 72 h.

While only a small portion of significant DE genes overlaps between MDM and HAM, we find a larger portion of highly expressed genes (>2,000 RPMs, ≥1.5-fold change) overlap between MDM and HAM (Table 1), while substantial differences remain. In total, 99 highly expressed genes were differentially expressed in at least one time point, in response to *M*.*tb* infection, with 18 DE genes highly expressed in all infected HAM and MDM across all time points (Table 1). Top Gene Ontology processes for these 18 highly expressed common genes include oxidative stress response, lipid antigen presentation, and endocytosis signaling. Up-regulation of *M*.*tb*-induced genes of some of these categories, especially those associated with oxidative stress response or respiratory burst, is consistent with other reports on expression changes upon *M*.*tb* infection in human macrophages, especially MDM [20, 22].

**Table 1:** Highly expressed (>2,000 RPM) DE genes in HAM and MDM at 2, 24, and 72 h post *M*.*tb* infection. The common and unique DE genes are separated out. DE genes are defined as those with ≥1.5-fold change of mRNA expression in either direction upon *M*.*tb* infection. These genes also meet the criterion of adjusted p = ≤0.05 by edgeR. ‘Common to all’ indicates that these genes are DE genes >2,000 RPM in both HAM and MDM at all three time points, while ‘common to all HAM’ and ‘Common to all MDM’ indicates overlap at all time-points only for HAM or MDM.

Unique 2 hr HAM > 2000 RPM	Unique 24 hr HAM > 2000 RPM	Unique 72 hr HAM > 2000 RPM	Unique 2 hr MDM > 2000 RPM	Unique 24 hr MDM > 2000 RPM	Unique 72 hr MDM > 2000 RPM	>2000 RPM common to all HAM time points	>2000 RPM common to all MDM time points
CDKN1A	CFL1	CCL18	ACP5	GBP1	CD14	ACTG1	CHI3L1
CST3	CLIC1	CYBB	CALM1	MT2A	G0S2	CD68	CHIT1
DDX5	CSF1		CCL22	STAT1	HLA-E	CXCL8	CTSB
DUSP1	CTSL		CD63		TNFAIP6	EIF4A1	CTSZ
EIF1	CXCL5		CD164			HLA-DRA	FCER1G
GADD45B	FLNA		VAT1			HLA-DRB1	GAPDH
H3F3A/H3F3B	IL1RN					S100A11	LIPA
HBEGF	SERPINB2					TFRC	MMP9
HLA-DPA1	TMBIM6					TPT1	SPP1
HSPA5	YBX1					UBB	**ACTB**
HSPA8						**ACTB**	**APOE**
HSPA1A/HSPA1B						**APOE**	**B2M**
PABPC1						**B2M**	**CTSD**
RPL4						**CTSD**	**EEF1A1**
RPLP2						**EEF1A1**	**FTH1**
RPS11						**FTH1**	**FTL**
						**FTL**	**GPNMB**
						**GPNMB**	**IFI30**
						**IFI30**	**LAPTM5**
						**LAPTM5**	**LYZ**
						**LYZ**	**MALAT1**
						**MALAT1**	**PSAP**
						**PSAP**	**RPPH1**
						**RPPH1**	**SOD2**
						**SOD2**	**TMSB10/TMSB4X**
						**TMSB10/TMSB4X**	**TYROBP**
						**TYROBP**	**VIM**
						**VIM**	

### IPA of DE genes: Networks and pathways

**Fig 4:**
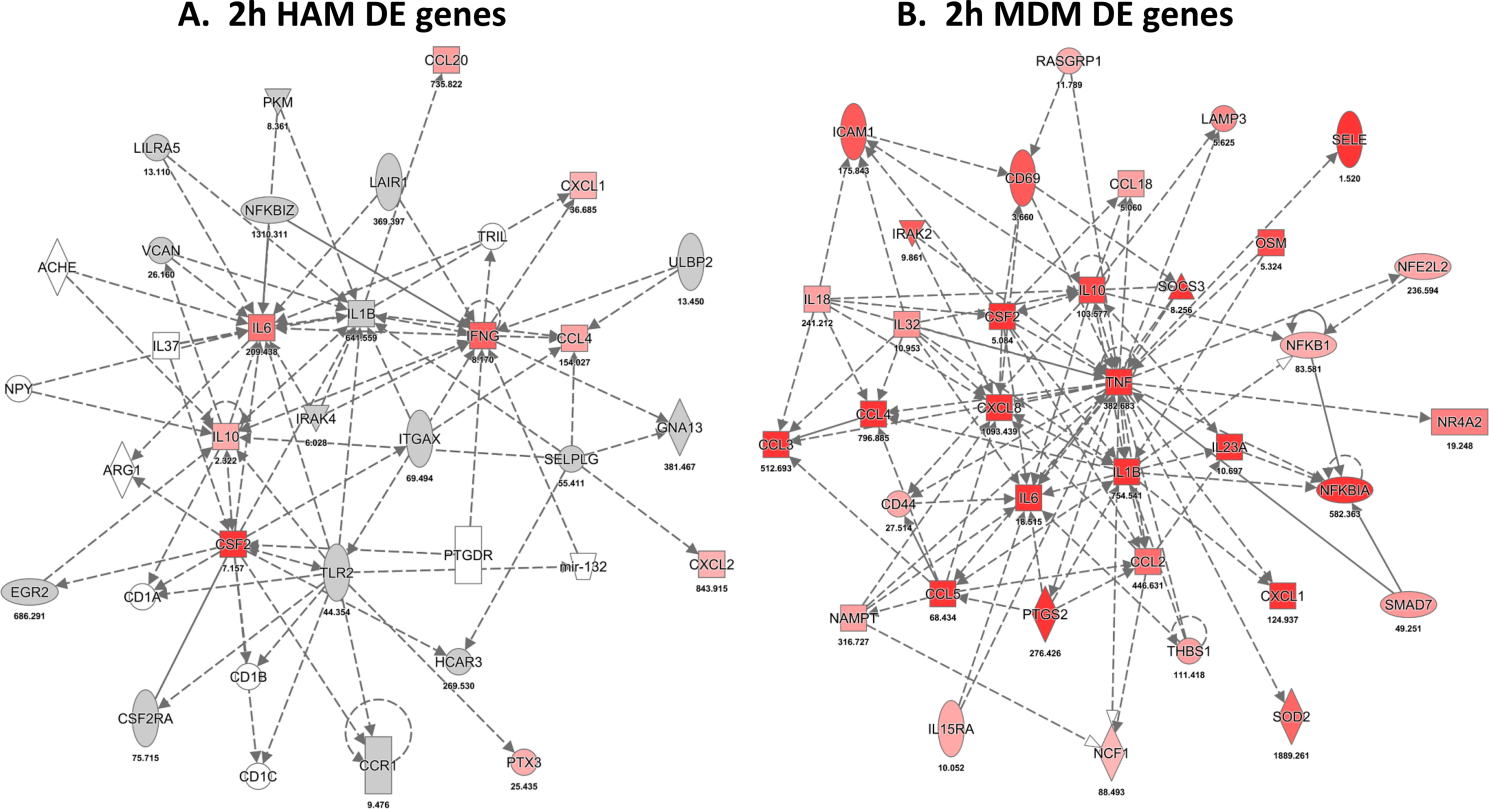
**Top scoring network of DE genes (IPA results) after 2 h upon *M*.*tb* infection in HAM (A) and MDM (B)**. Red indicates mRNA up-regulation and green down-regulation. Intensity of color is proportional to log2 differential expression ratio (DER) of average paired infected/control RNA expression. DE ratios used are the mean of three HAM and three MDM donor samples.

Gene network analysis based on known molecular interactions (IPA) (https://analysis.ingenuity.com/) defines molecular patterns that provide insight into the *M*.*tb*-macrophage interactions. The edgeR statistical method served to select DE genes for HAM and MDM at each time point (mean cutoff ≥2-fold change and adjusted p≤0.05). Designed to identify primary regulators, based on statistical probability, IPA revealed patterns of gene expression changes in response to *M*.*tb* exposure that differ between MDM and HAM, especially at the earliest time point. The top IPA network of DE genes in both macrophage cell types at 2 h (Cellular Movement, Hematological System Development and Function) is shown in Fig 4A and 4B. Within 2 h, MDM respond to *M*.*tb* infection with an up-regulation of cell surface receptors involved in antigen presentation (CD40, CD83, TNFRSF9), C-lectin and lymphocyte proliferation-linked receptors (CD69, CD80, CD3e), and signaling pathway and cytokine/chemokine transcripts (CCL3, CCL4, CXCL8, IL-1B, IL-10, IL-21, IL-2RA). In contrast, the initial HAM response to infection displays a lag phase with few genes significantly up-regulated at the 2 h post-infection time point (CSF2, IL-1B, IL-6, IL-10, IL-21). IL-10 is a hub in both HAM and MDM at the 2 h time point.

**Fig 5:**
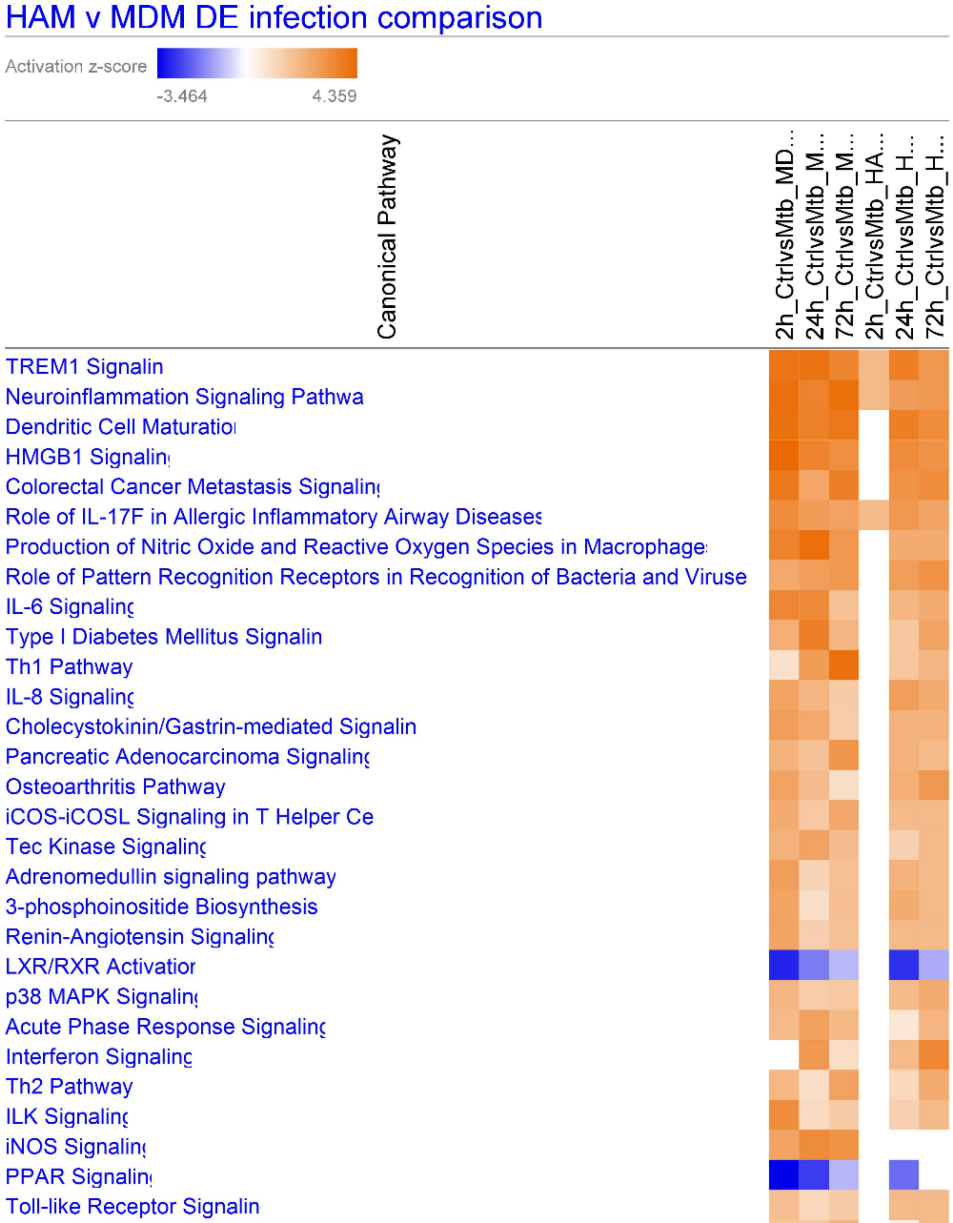
IPA heat map of canonical pathway activation in HAM and MDM upon *M*.*tb* infection at 2, 24 and 72 h, derived from DE genes with adjusted p≤0.05. Colors represent activation z scores. TREM1 signaling is the top pathway in both MDM and HAM. Columns left to right are: 2, 24, 72 h MDM, 2, 24, 72 h MDM infected.

We next addressed differences in canonical pathways of response genes (DE genes with adjusted p≤0.05) (IPA) after *M*.*tb* infection of HAM and MDM from 2–72 h. Shown in Fig 5, the lagging response of HAM at 2 h is clearly visible, while differences are less pronounced at the later time points. TREM1 signaling is the top pathway across all samples, although more prominent in MDM. Other pathways more robustly expressed in MDM over HAM include ‘production of nitric oxide and reactive oxygen’, ‘Th1 pathway’, and ‘inflammasome pathway’, whereas ‘interferon signaling’ is more pronounced in HAM at 72 h. Notably, the LXR/RXR and PPAR pathways are down-regulated in both macrophage types. Further delineation of specific genes in the pathways and the specific transcription factors await further study.

### *TREM1* gene cluster and pathway

Triggering Receptor Expressed on Myeloid Cells 1 (TREM1) is a top scoring canonical pathway in both HAM and MDM infected with *M*.*tb*. TREM1 amplifies myeloid cell inflammatory responses by stimulating the release of cytokines and cell surface activation markers. Although TREM1 signaling is a top pathway and up-regulated at all time-points, specific gene response varies between HAM and MDM as well as from individual to individual. A key gene in immune and inflammatory responses, *TREM1* resides in a gene cluster of several *TREM* homologues, spanned by long linkage disequilibrium (LD) blocks indicative of evolutionary selection pressure (S1 Fig). Moreover, the region is studded with numerous highly significant eQTLs in whole blood, available from GTEx, that also extend over the entire gene cluster. SNP rs4714444 has the lowest p value for *TREM1* in whole blood (GTEx), but is also an eQTL for 3 other *TREM* genes in the same cluster (color coded dots in S1 Fig). This result indicates that genetic factors in the regulation of gene expression in such a multi-gene cluster influence the entire region with inter-dependent mechanisms.

**Fig 6:**
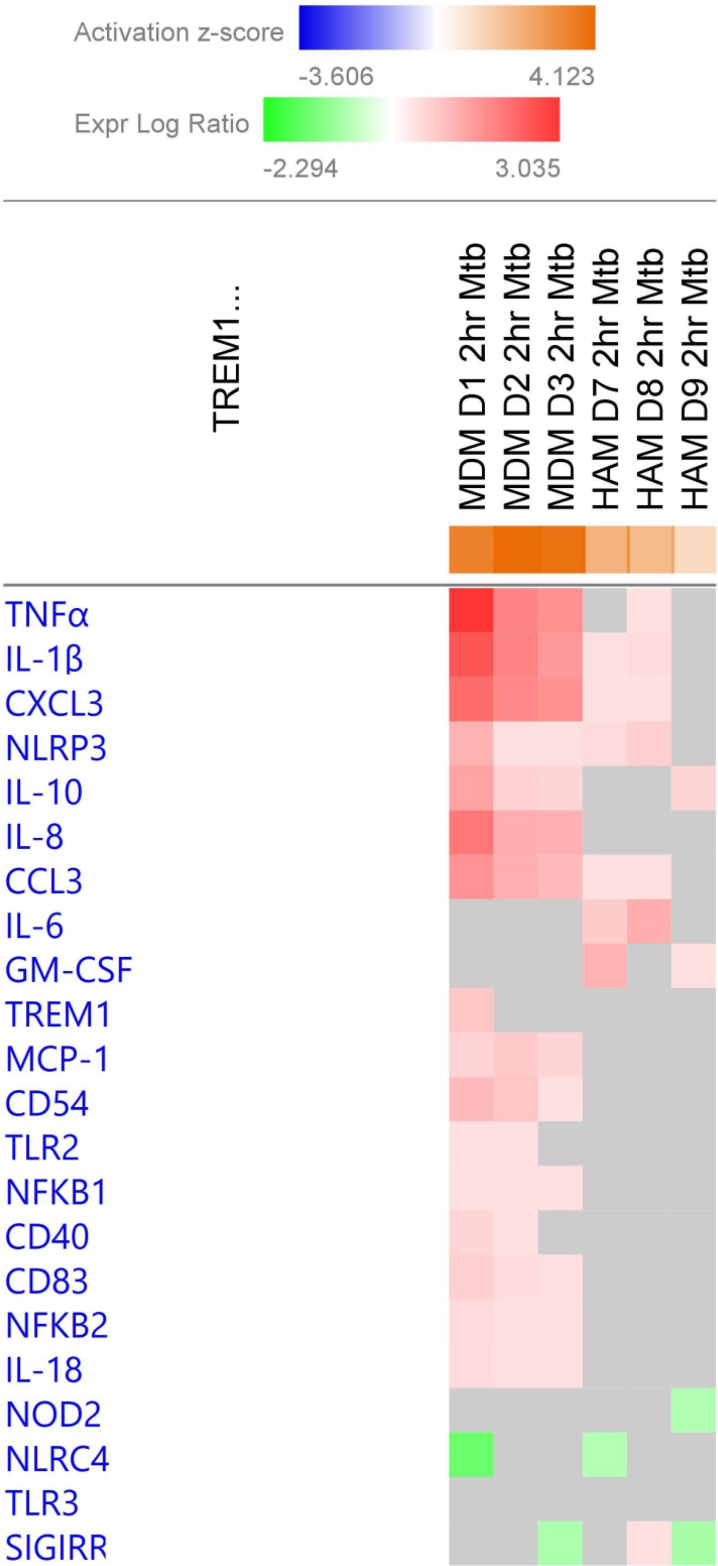
TREM1 pathway gene activation in individual HAM and MDM samples at 2 h post *M*.*tb* infection. The heatmap color and intensity indicate the ratio of mRNA expression *M*.*tb*/control cells. Red indicates a positive activation z-score and blue is a negative z-score. IL-6 and GM-CSF (CSF2) expression stand out in all 3 HAM samples at 2 h whereas multiple other genes are more highly differentially expressed in MDM.

Molecules in the TREM1 pathway are activated at all time-points in both HAM and MDM. A more detailed view of DE genes in the TREM1 pathway at 2 h across the three donors each for both types of macrophages is shown in Fig 6. The top-scoring DE genes are found in MDM, mostly with uniform stimulation across all three donors, except for TREM1 mRNA itself showing up-regulation only in donor D1. In contrast, *M*.*tb* stimulation is lower in HAM for most genes, except for GM-CSF (CSF2) and IL-6, also with large inter-individual variability. This result indicates that rapid response in HAM does occur but is limited to a smaller set of genes that in part differ from those in MDM at this early time point. GM-CSF in particular is a critical driver of HAM differentiation [46–48]. Defects in the GM-CSF pathway are associated with alveolar proteinosis and a propensity for mycobacterial infections [49]. Further interpretation of our results will require protein analyses in a larger sample set.

### *IL-10* gene locus, regulatory variants, and association with resistance to TB

**Fig 7:**
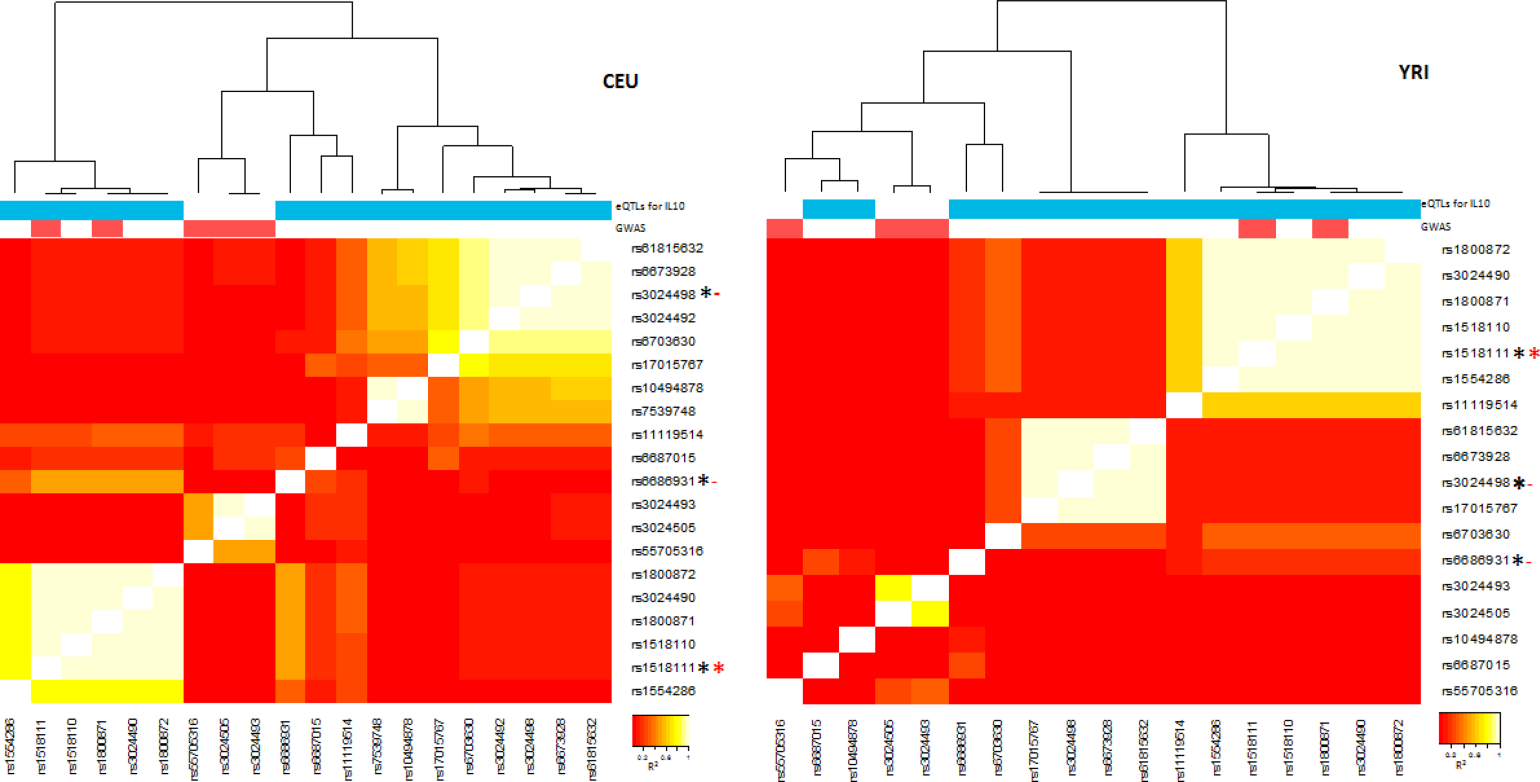
Linkage disequilibrium or LD heatmap of SNPs around the *IL-10* gene locus that are either recognized as eQTLs in GTEx or as GWAS hits in dbGaP (for any phenotype), or both. LD maps are constructed from Caucasian (CEU) and Yoruban (YRI) genomic SNP data, showing different haplotype configurations. The colored bars on top mark SNPs with eQTLs for IL-10 mRNA (blue), and dbGaP hits (red). The three marker SNPs highlighted (rs1518111, rs6686931, rs3024498) are marked by a * if recognized as an eQTL (black) or a dbGaP hit (red). Only rs1518111has both associations along with other variants in its LD block (white squares), while rs6686931and rs3024498 are only eQTLs. rs6686931 is in partial LD with rs1518111 in CEUs but in lower LD in Yorubans (YRIs). GWAS variants are part of two separate blocks—one separate from all eQTLs and one part of the LD block marked by rs1518111.

*IL-10* is a hub gene in immune response networks in both MDM and HAM. IL-10 is a key regulatory cytokine dictating host susceptibility to TB in humans and mice [50–54]. The *IL-10* gene locus has multiple GWAS hits, including association with inflammatory and autoimmune disorders, including Behcet’s Disease (rs1518111, p = 4e-18) [51, 55–57], and with numerous eQTLs specific for IL-10 mRNA expression in GTEx, distributed over a large genomic region. Data from GTEx and Haploreg indicate that there are three distinct overlapping long LD blocks affecting regulation of IL-10 RNA transcripts (Fig 7). The top scoring eQTLs in each of the three LD bins exist at relatively high but varying frequencies in different ethnic populations. The top scoring SNPs for each LD bin include rs1518111, rs6686931, and rs3024498, each with similar eQTL p values. Even though rs6686931 (r^2^<0.5) and in particular rs3024498 (r^2^<0.1) are in relatively low LD with rs1518111, both score with eQTL p values <e-5, suggesting the presence of 2–3 independent regulatory variants with different mechanisms of IL-10 transcript regulation. Distinct from the *TREM* multi-gene cluster with potentially multiple regulatory variants for multiple genes, here we find multiple regulatory variants converging on a single gene.

The LD heat map of SNPs with significant eQTL p values and GWAS hits (any phenotype) across the *IL-10* gene locus, shown in Fig 7, displays the relationship between the three signature SNPs in distinct LD bins with multiple other SNPs in high LD each. Moreover, the LD pattern substantially differs between Europeans (CEU) and Africans (YRI). Of the three signature SNPs, only rs1518111 coincides with a GWAS-significant SNP (Fig 7). Since the other GWAS hits also do not reside in protein-coding exons, these are likely to be regulatory as well, but are not recognized as blood eQTLs in GTEx, possibly because of association with RNA isoforms or tissue-specific effects.

We then extracted the top scoring SNPs within the *IL-10* region from an association study of *M*.*tb* infection obtained from a household contact study conducted in Uganda [4, 43, 58]. Nine SNPs score with p <0.01 (Table 2), including rs518111. Variants affecting IL-10 transcripts have differing prevalence in different populations, so that the LD structure and haplotypes will also differ–a confounding factor that must be accounted for in TB-gene association studies across populations. Specifically, rs1518111 is present at high frequency in a strong LD block in sub-Saharan Africans (YRI) (Fig 7, right panel). Moreover, the association between rs1518111 and Behcet’s disease [55, 57] may be due to an altered expression of transcript isoforms. rs1518111 resides in an intronic region of the reference IL-10 mRNA and is a strong eQTL in GTEx (p = 2.5e-9).

**Table 2:** Association of *IL-10* SNPs with persistent TST negative (PTST–) phenotype. The most significant SNPs in the *IL-10* gene plus 100 kb upstream and downstream are listed.

			EA Frequency			* *
SNP	Chr. 1 BP	Alleles	PTST–	Not PTST–	OR	*p*
rs1554286#	206,944,233	T/C	0.45	0.36	1.45	0.0092
rs1518111*#	206,944,645	A/G	0.45	0.36	1.45	0.0092
rs13208	206,828,162	T/C	0.32	0.41	0.69	0.0063
rs73079064	206,714,842	T/C	0.24	0.31	0.66	0.0078
rs11119107	206,767,938	A/G	0.16	0.09	1.72	0.0082
rs12075916	206,765,672	A/G	0.10	0.17	0.54	0.0024
rs12077772	206,777,225	A/G	0.08	0.15	0.45	0.0010
rs12079722	206,765,254	T/C	0.08	0.13	0.52	0.0068
rs17012590	206,779,884	C/T	0.02	0.05	0.31	0.0062

EA Frequency, frequency of effect allele; Alleles, effect/other allele; OR, odds ratio for PTST–

*, also a GWAS hit

#, also an eQTL in GTEx.

These two examples, *TREM1* and *IL-10*, illustrate the complexity of genes and gene clusters with multiple interacting genetic variants with biological significance. These all must be identified, and the resultant haplotypes in various populations determined before a correct assessment of the genetic influence of these gene loci can be estimated.

## Discussion

This study tests the utility of a method for transcriptome analysis, called AmpliSeq, measuring 18,574 mRNAs and 2,228 ncRNAs, to detect DE genes induced by *M*.*tb* in human macrophages [30]. The assay precision enables sensitive detection of DE genes when comparing uninfected control and *M*.*tb*-infected cells obtained from healthy volunteer donors. Applying AmpliSeq to MDM and HAM, both baseline and exposed to *M*.*tb*, resulted in highly reproducible results.

A second goal of this study was a preliminary assessment of the transcriptome response in human macrophages to *M*.*tb* infection, and to compare the response between HAM, a primary target and niche for *M*.*tb* in the lung, and MDM–a commonly used *in vitro* human macrophage model of *M*.*tb* infection. While overall number of detectably expressed genes is similar between HAM and MDM (Fig 2), baseline RNA profiles differ substantially, particularly at 2 h incubation, including genes relevant to TB pathophysiology (S1 Table). The unique gene expression profile of freshly obtained HAM wanes over time in culture as adherent cells. For example, a subset of RNAs (especially matrix metalloproteases MMP7 and MMP9) are initially low in HAM but high in MDM, yet increase over time as HAM are cultured as monolayers. Taken together, a number of baseline HAM RNA levels at 72 h begin to resemble those in MDM and may reflect an adherence phenotype, but substantial differences remain even at 72h.

Measuring the time course of gene expression in 3 MDM and 3 HAM donor samples in controls and after *M*.*tb* infection yielded deep profiles of DE genes (S1 and S2 Tables). Details can be viewed in the interactive table (https://dataportal.bmi.osumc.edu/macrophagedb/). As a result of the precision of AmpliSeq, a large number of DE genes are detectable with high significance (S2 Table), even with only 3 individuals for each macrophage type analyzed, yielding results that are generally consistent with previous reports [14–20, 22, 23], using hybridization chips or full RNA-Seq. In addition, several DE genes also overlap with those obtained from a mouse model following infection of iNOS-deficient and WT mice with *M*.*tb* strains (24). Tailleux *et al*. [59]measured RNA profiles with microarrays in *M*.*tb*-infected MDMs compared to dendritic cells, identifying common and MDM-specific pathways that may account for the more permissive cellular environment in macrophages, including production of IL-1B and IL-6, as was found here. Interestingly, using the microarrays, Tailleux *et al*. report that donor-to-donor variability only weakly influences the RNA response profiles, which differs from our observation of substantial differences for individual genes, measured with AmpliSeq [59]. Indeed, detecting inter-individual differences is one important long-term objective of our studies to characterize individual immune response pathways that reflect differences in susceptibility to *M*.*tb*.

Striking differences exist in RNA expression between HAM and MDM in the number of DE genes and the degree of stimulation or suppression. Maintained in *in vitro* culture with autologous serum for 6 days, MDM express inflammatory factors more strongly and respond to *M*.*tb* more rapidly and robustly than HAM, most obvious at 2 h when the response of freshly harvested HAM is small. HAM are used within 6 h outside the body, and over the 72 h incubation period, they increase expression of a number of the same inflammatory factors observed in MDMs, but only to intermediate levels. *In vitro* culture conditions appear to mobilize MDM inflammatory defense responses more strongly over those of the freshly obtained HAM. Taken together, we posit that freshly obtained HAM are still conditioned at 2 h by the *in vivo* environment. While we do not know the exact *in vivo* HAM-*M*.*tb* conditions, these results highlight the influence of *in vitro* culture conditions on RNA expression profiles, and thus the *M*.*tb*-macrophage interaction.

Applying IPA to the datasets served to assess pathways and gene networks in uninfected control and *M*.*tb*-infected HAM and MDM. Uninfected control cell pathways display significant differences between HAM and MDM, while the sample size is too small to evaluate differences in expression of individual genes because of substantial variability between subjects [with the exception of a few genes with extreme differences (S1 Table)]. However, we note that the baseline RNA profiles of HAM–initially in a less active state–evolve to resemble the more active MDM over the 72 h incubation even in the absence of *M*.*tb*, further highlighting that the *in vitro* culture alone is a significant stimulant.

Focusing on DE genes, the IPA results reveal overlapping and distinct canonical pathways (using IPA) between HAM and MDM, with the TREM1 pathway prominent in both macrophage types (Fig 6). Only a few pathways are detectably stimulated by *M*.*tb* at 2 h, but subsequently, HAM and MDM display similar pathways. The immediate HAM response at 2 h is limited to a few strongly activated genes [IL-6, IL-10, IL-21, IL-1B, and CSF2 (GM-CSF)], whereas the MDM network hub genes reveal a much stronger inflammatory response (IL-1B, IL-6, IL-10, IL-21, IL-2RA, CXCL8, PTGS2, and more). As noted earlier, this difference between HAM and MDM diminishes over time to 72 h.

We observe substantial differences between individuals in both HAM and MDM, consistent with reported large inter-individual differences in uptake and growth of *M*.*tb* in these macrophages [9, 11, 22, 60], and specifically in HAM [18, 23, 25, 45]. Since the genetics of TB susceptibility remain poorly understood, a third goal of this study was to lay the groundwork for an approach that identifies those genes differentially expressed upon *M*.*tb* exposure with evidence of carrying regulatory variants that affect their expression. This approach will be used in a larger study to identify genetic differences between HAM donor responses that could presage susceptibility to TB. In particular, genes of the innate immune system have accumulated genetic variants as a result of evolutionary pressures [9], the vast majority of which are likely to be regulatory (affecting transcription and RNA processing or functions) [13].

To highlight next steps from response gene networks to genetic factors, we evaluate two response network hub genes, namely *TREM1* and *IL-10*, the former emerging in both HAM and MDM as a top inflammatory signaling pathway [61–63], and the latter previously identified as a cytokine with a critical role in controlling inflammatory processes [17, 64, 65]. *TREM1* exemplifies a gene residing in a large cluster of homologues, covered with long LD bocks with frequent SNPs, an indicator of evolutionary selection pressures (S1 Fig). The gene cluster is decorated with numerous eQTLs (GTEx), many of which appear to interact with more than one gene in the cluster–possibly via chromatin looping among multiple enhancers and promoters [66, 67]. We find numerous such multi-gene clusters among genes of the innate immune system that require combined analysis of the genetic influence of the entire cluster rather than that of single SNPs acting independently, as shown for a nicotinic receptor gene cluster [68].

On the other hand, *IL-10* resides alone in a gene region also showing multiple eQTLs in blood (GTEx) that are all specific for IL-10 expression. Moreover, dbGAP reveals a series of significant GWAS hits in and around *IL-10* (Fig 7), showing SNPs associated with Behcet’s disease and ulcerative colitis, some overlapping with *IL-10* eQTLs [54–57]. For example, rs1518111 is such an eQTL with strong GWAS hits, suggesting a functional relationship of this SNP, or a variant in high LD with it, between expression and clinical association. Since this SNP resides in an intron, it may be associated with an RNA isoform or it could also be in high LD with another variant affecting RNA expression or processing. The pattern of eQTL p values and LD data across the *IL-10* locus (Fig 7) suggests the presence of several regulatory variants that act independently of each other and show distinct minor allele frequencies (MAFs) across ethnic groups. To assess accurately the genetic influence of such complex gene loci, haplotypes constructed from the main causative variants are best suited to assess the combined genetic influence of each gene locus [68]. These can now be efficiently detected with ultra-long-read sequencing technologies [69].

Taken together, our study uses AmpliSeq to highlight distinct features in gene expression between MDM and HAM and emphasizes the influence of in vitro culture conditions on gene expression. At the same time these 2 macrophage cell types also demonstrate several pathway similarities in their responses to *M*.*tb* infection. Acknowledging these similarities and differences, MDM serve as a suitable model of *M*.*tb* interaction with primary human macrophages. Ongoing studies are addressing factors that allow for MDM to be cultured in ways to better recapitulate HAM. As a second feature of our study we propose a path forward using macrophage genetic analyses to gain better understanding of genetic susceptibility to TB.

There are limitations to our study. First, the small number of subjects precludes full description of the range of inter-individual cellular response to *M*.*tb* infection, but use of a highly reproducible RNA assay (AmpliSeq) enables DE gene discovery from small sample numbers mitigating this limitation to some extent. Further, the three HAM and MDM donor samples each were obtained from different individuals, precluding a direct comparison between MDM and HAM from the same individual. Nevertheless, work done for many years in our laboratory (Schlesinger) shows that individual healthy donors have consistent biological responses to *M*.*tb* and other antigens when studied over time. The results presented here will prove valuable in a larger follow-up study when analyzing HAM from donors with extreme responses to *M*.*tb*.

## Supporting information

S1 Table Genes highly expressed at 2 h only in either HAM or in MDM. For genes highly expressed in HAM at 2 h, we also list expression levels at 72 h in HAM, showing the changes during incubation. The 72 h levels are not provided for MDM because changes are mostly minimal. Relative expression levels are in RPMs. Genes previously implicated in TB biology are highlighted (asterisked).
(XLSX)

S2 Table Searchable dataset of RNA expression in HAM and MDM, providing detectable genes, RNA expression levels (Reads Per Million, RPMs) of the mean RPMs of controls plus *M*.*tb*-infected, the fold-change between control and *M*.*tb*, the p value of differential expression (edgeR), and the false discovery rate (FDR). Genes with and adjusted p value of ≤0.05 are included, except for DE genes in HAM at 2 h (≤0.10). Complete data will be made available in the NCBI Gene Expression Omnibus.
(XLSX)

S1 Fig *TREM1* gene cluster with expression quantitative trait loci (eQTLs) in whole blood from GTEx. Grey dots represent eQTLs for any of the genes in the cluster [go to GTEx to identify the target gene(s)]. Shown in 4 different colors is rs471444, the highest scoring blood eQTL for *TREM1* (light blue) also is an eQTL for three additional *TREM* genes highlighted in different colors.
(TIF)
