# Supplementary figures and images for: AmpliSeq transcriptome analysis of human alveolar and monocyte-derived macrophages over time in response to Mycobacterium tuberculosis infection
Source: PLoS One. 2018 May 30;13(5):e0198221. doi: 10.1371/journal.pone.0198221 (PMC5976201; doi:10.1371/journal.pone.0198221)

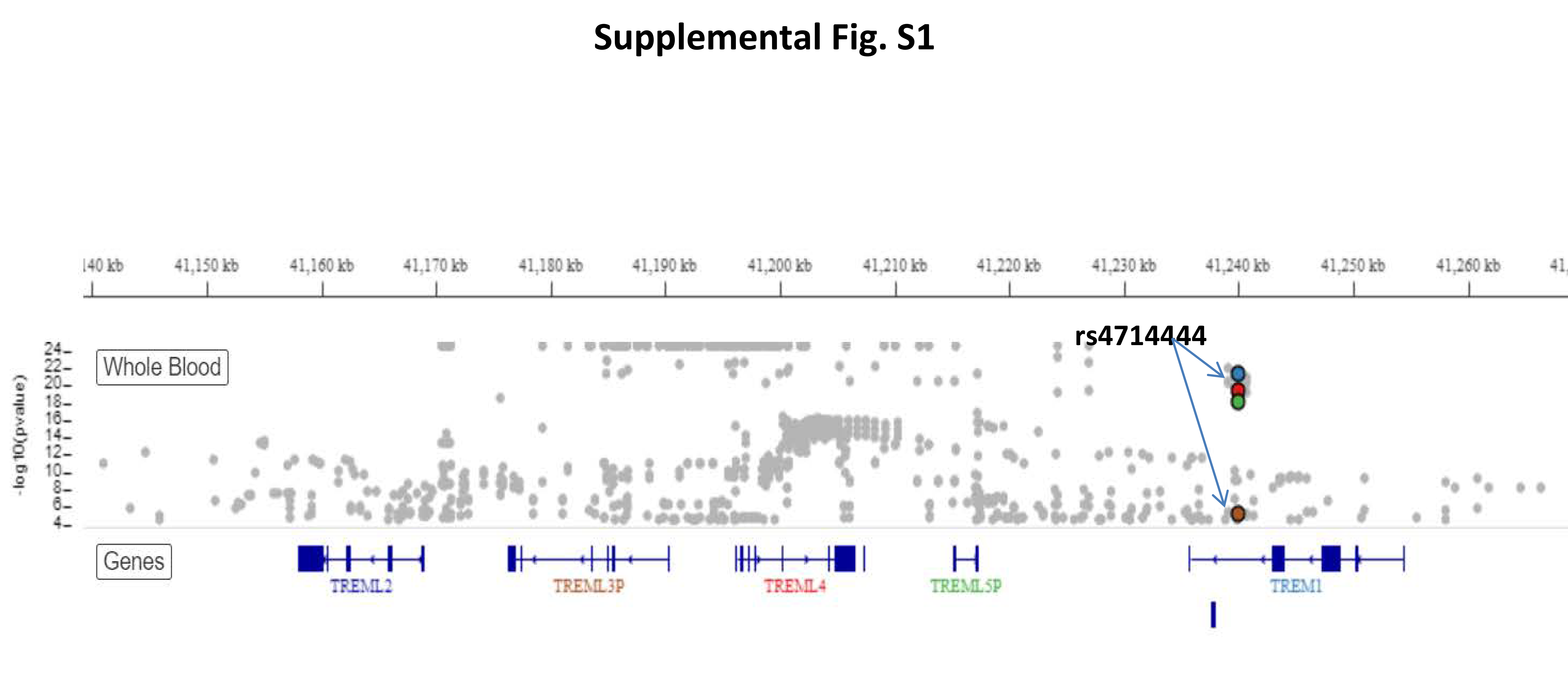

Supplement: S1 Fig — Grey dots represent eQTLs for any of the genes in the cluster [go to GTEx to identify the target gene(s)]. Shown in 4 different colors is rs471444, the highest scoring blood eQTL for TREM1 (light blue) also is an eQTL for three additional TREM genes highlighted in different colors. (TIF) [file pone.0198221.s003.tif]
